# Determinants of consent for electronic health information exchange: an observational retrospective study

**DOI:** 10.1186/s12961-025-01377-x

**Published:** 2025-08-07

**Authors:** Jelle Keuper, Karin Hek, Lilian H. D. van Tuyl, Ronald Batenburg, Robert Verheij

**Affiliations:** 1 Netherlands Institute for Health Services Research, Utrecht, Netherlands; 2 Tranzo, Tilburg School of Social and Behavioral Sciences, Tilburg University, Tilburg, Netherlands; 3 Department of Sociology, Radboud University Nijmegen, Nijmegen, Netherlands; 4 National Health Care Institute, Diemen, Netherlands

**Keywords:** General practice, Electronic health records, Health information exchange, Routine health data, Consent, Opt-in

## Abstract

**Background:**

Sharing of patient electronic health record (EHR) data between healthcare providers can enhance quality and efficiency of healthcare provision, and patient safety. Health information exchange is allowed only with explicit patient consent. In the Netherlands, patient consent and the exchange of information is organized nationally. Consent status is recorded routinely in general practice EHRs. This study examines how various characteristics at the individual and general practice level influence this consent for electronic health information exchange (HIE).

**Methods:**

Routine EHR data from general practices and out-of-hours primary care services participating in the Nivel Primary Care Database were analysed for the period 2017–2019, just before the coronavirus disease 2019 (COVID-19) pandemic outbreak. Bivariate chi-squared test analysis and multilevel logistic regression analysis, adjusted for practice-level clustering, were conducted for each of the 3 observed years to assess whether consent for electronic HIE (“yes” or “no”) was associated with the individual’s health and healthcare utilization, demographics, neighbourhood characteristics (socioeconomic position, degree of urbanization, area deprivation), and general practice characteristics (practice type, EHR system, practice size).

**Results:**

Between 2017 and 2019, 38–45% of the individuals provided consent for electronic HIE. Individuals with a higher number of different prescriptions or those with long-lasting health problems or chronic diseases had lower odds of providing consent, in each of the 3 years. This result also applied to female and older individuals (aged ≥ 65 years). In contrast, individuals from below-average socioeconomic position neighbourhoods living in deprived urban or hardly urbanized regions generally had higher odds of providing consent.

**Conclusions:**

In contrast to what was expected, patient groups with higher healthcare utilization were less, or as likely to provide consent for HIE compared with individuals with no or lower healthcare utilization. This implies that the population most likely to benefit from HIE is, in fact, less likely to profit from it. Further research is needed to determine whether these differences arise from individual trust, privacy concerns, transparency issues or other factors such as physicians’ HIE beliefs. Optimizing the national HIE system in the Netherlands requires considering multiple influencing factors, on both the individual level and practice level.

**Supplementary Information:**

The online version contains supplementary material available at 10.1186/s12961-025-01377-x.

## Background

Globally, medical and health data from citizens and patients are increasingly generated and stored electronically. These data generally include biometric data from personal devices, including smartphones (apps) or wearables, and data recorded routinely during the healthcare process in the form of electronic health records (EHR) and claims data, pertaining to health problems, prescription drugs and lab test results [1]. Through electronic health information exchange (HIE) systems, healthcare providers are facilitated in accessing up-to-date individual patient health data. This is generally known as primary use of health data. HIE encompasses the process of appropriately accessing and securely sharing patient-level medical information (electronically) across a network of actors, including family physicians, pharmacists, medical specialists and patients [2]. Expected benefits of improved HIE include healthcare process efficiencies, reduced healthcare costs and improved quality and continuity of care, a better understanding of a patient’s health and better patient safety and health outcomes (e.g. by avoiding medication errors, improving diagnoses and decreasing unnecessary duplicate testing) [1–6]. However, these benefits can only be experienced if the electronic HIE infrastructure functions properly and is well integrated within healthcare organizations. Furthermore, legal and financial challenges may be dealt with by the actors involved [7]. A systematic review by Eden et al. [8] investigated important barriers and facilitators to HIE use, including (in)complete patient information, (in)efficient organization and workflow, technology and user needs [8].

The adoption and use of HIE is widespread and differs considerably between countries, and its implementation has been investigated extensively in many countries during the last two decades, especially in the United States and several European countries. In the Netherlands, the Ministry of Health already opted for a national HIE system in the mid-1990s [7, 9, 10], misleadingly called the National EHR (landelijk elektronisch patiëntendossier). The term National EHR led to misunderstandings in the general public because it did suggest national storage of data in one facility, which was not the case, as data were stored locally within each healthcare provider’s domain. Eventually, the proposal for this national system was voted down unanimously in the Dutch Senate in 2011 owing to concerns about privacy, security and control over medical data. The central government stepped back from organizing the exchange of health data, which gave way to private regional and sectoral initiatives with often equally insufficient privacy safeguards [7]. A new attempt to establish a national system for HIE called the National Exchange Point (Landelijk Schakelpunt (LSP)) was made in 2012, facilitated by the Association of Healthcare Providers for Healthcare Communication (Vereniging van Zorgaanbieders voor Zorgcommunicatie (VZVZ)). This LSP system allowed healthcare providers to share individual patient data electronically on a national level. Similar to the previous National EHR system, this LSP system did not involve mass storage of data in one facility; it only facilitated the exchange of data stored locally across the healthcare system.

Healthcare providers are not obliged to participate in this system and are only allowed to access and exchange patient health information from patients who explicitly provided consent (i.e. opt-in) and when it is needed for their treatment [11, 12]. This opt-in system for primary use of patient health data, which also applies in a similar way in Germany and some other countries, differs from opt-out policies used in countries such as Austria, the United Kingdom, Scandinavian countries and in some health organizations in the United States. In opt-out systems, by default, citizens’ health data are stored and shared among healthcare providers for treatment purposes, but individuals can restrict access to their medical records by opting out. This generally results in higher participation rates [8, 13–15]. In the Netherlands, there are exceptions to the national electronic opt-in system, referred to as “push” HIE. In these cases, explicit consent from the patient is not required. Instead, patient consent is presumed, though patients retain the option to object. Examples of “push” exchanges include the use of electronic messaging between healthcare providers, such as lab results or letters from medical specialists, as well as patient referrals from general practices to medical specialists or health information transfer systems. In these instances, the initiative to exchange health information lies with the sender of the patient health information (e.g. the general practitioner (GP)) [16]. This study, however, focusses on the National Exchange Point opt-in system in the Netherlands. This system can be considered a “pull system”, enabling healthcare providers at primary care out-of-hours (OOH) services, for instance, to access a patient’s general practice EHR.

Within Europe, work is currently being carried out on a European Health Data Space (EHDS), which regulates control of patients’ electronic personal health data, at both the national level and European Union level [17]. The political agreement that has been reached on this is based on an opt-out system, which differs from the current National Exchange Point opt-in system in the Netherlands. However, there remains room for member states to choose an alternative option. Furthermore, for certain types of health data, an opt-in approach may be chosen instead.

In the Netherlands, consent can be provided by verbally indicating this at the individual’s GP and/or pharmacist or by filling out a consent form for HIE at their general practice and/or pharmacy. Then, the GP or pharmacist can register the citizen service number of consenting citizens with the National Exchange Point, through which other healthcare providers can access patient health data. Since 2017, citizens can also provide consent online at volgjezorg.nl, where they can also find an overview of healthcare providers that looked into their medical data [18]. In reaction to the COVID-19 pandemic, the government introduced a temporary regulation in April 2020, allowing for HIE between daytime general practices, primary care OOH services, and emergency care services for patients suspected of having a COVID-19 infection, who had not (yet) indicated whether they provided consent for HIE. In April 2023, this temporary measure stopped, and the former opt-in system was restored. Similar to the period before the pandemic, this is a two-tier system in which general practices must first register with the health data exchange platform before patients can give consent for the actual exchange of their data [19].

Whether an individual provides consent for HIE may depend on several factors. A literature review by Esmaeilzadeh et al. [20] indicated several important factors which affect the patient’s attitude towards HIE, including patient characteristics, patient participation level in HIE and perceived benefits [20]. A more recent systematic review by Cascini et al. also found that patient attitudes regarding sharing of their health data varied significantly across sociodemographic factors and depended on data type and type of use [21]. Other studies found that the mechanism used for HIE, privacy concerns, security risks, trust issues and psychological risks also influence the patients’ opt-in intention [22–30]. A study by Shi et al. [26] found that patients’ willingness to share their health data was positively associated with having access to complete information and medical error reduction, while no effect of age, gender, education level, income, perceived health status or place of residence on willingness to share health information was found [26]. Further, healthcare providers’ experiences with, and perceptions towards, the use of an electronic HIE system may also affect a physician’s choice to offer or promote the opt-in option for HIE to their patients, thereby influencing the possibility or willingness of a patient to provide consent [8, 31, 32].

The abovementioned studies, which mainly used surveys and qualitative study designs and focused on patient or professional attitudes, show that many patient characteristics are important in patients’ willingness to provide consent for electronic HIE. How factors such as healthcare utilization and other individual and general practice-related factors were important within the context of the national HIE opt-in system in the Netherlands remains unknown. Owing to the temporary regulation implemented during the pandemic years, the recent restoration of the pre-COVID situation, and the unavailability of data on the post-COVID years, we decided to focus on the pre-pandemic period.

As mentioned before, previous studies that focused on people’s inclination or intention to consent to HIE were based on surveys or interviews, making the results prone to selection bias and telescoping. Furthermore, studies often focus on patients rather than citizens or the general population. In this study, however, we focus on actual opt-in behaviour, using real-world data from general practice EHRs, in which opt-in status is routinely recorded for individuals who do and do not utilize health services within a system where virtually every citizen is enrolled in a general practice. The latter aspect ensures that, with the data we use, we are essentially dealing with a population sample rather than a sample of patients. Moreover, data linkages and the longitudinal character of the EHR data we used for our analyses make it possible to consider the complete medical history of patients. Moreover, the data allow us to systematically investigate practice variation, and clustering in practices, providing opportunities to investigate the role of general practices in the possibilities of HIE. In addition, individual-level and practice-level characteristics associated with providing consent for electronic general practice HIE can be identified. To the best of our knowledge, this is the first study investigating people’s actual consent behaviour, using routinely recorded health data.

## Methods

### Study design and setting: routinely recorded health data

For this observational retrospective study, we used pseudonymised EHR data from 2017 to 2019, recorded routinely in general practices and primary care OOH services that participated in Nivel Primary Care Database (Nivel-PCD) [33]. This pertains to a sample of up to 10% of (approximately 5000) Dutch general practices and 58% of (approximately 50) primary care OOH services. The patient population within these databases closely reflects the Dutch population in terms of age and gender distribution. For the primary care OOH services, individuals from highly urbanized areas are slightly overrepresented, while those from less urbanized areas are underrepresented. The Dutch general practices participating in Nivel-PCD are also representative with regard to most of the practice-level characteristics, with only an underrepresentation of duo practices and practices in both not-urbanized and highly urbanized areas. Within these databases, we distinguished practice- and individual-level variables. Because of the two-tier structure of the National Exchange Point (see above), consent registration data were only available from general practices connected to the system. This was the case for the majority of practices during the study period [34]. The studied consent applied only to the exchange of health information by healthcare providers through this national HIE opt-in system. Although regional HIE systems do exist, this national system is endorsed by national policies and is considered as the most important, facilitating the secure exchange of information between healthcare professionals.

### Measures

The study’s outcome measure is citizen consent for electronically sharing general practice health data with other healthcare professionals who may need to access these data for a patient’s treatment. This outcome measure was routinely recorded as either “yes” or “no” in the general practices’ EHRs. Here, “yes” indicates explicit consent has been provided by an individual, while “no” means the individual has either not given explicit consent or has explicitly refused the exchange of their health information. For a selection of general practices and their patients, consent registration data were also available from 2015 and 2016. If an individual’s consent registration was missing in the databases in 1 of the following years, but was known in 2015 or 2016, the missing registration was replaced by the last known value in 1 of these previous years. Herewith, our assumption was that an individual’s consent remained the same in the following years if no change to “yes” or “no” had been recorded. This was also applied in 2018 and 2019, if an individual’s consent was not available in these years but had been recorded in an earlier year.

For the independent study variables, we distinguished four categories of variables to describe groups of related factors influencing consent, encompassing (1) individual health and healthcare utilization, (2) demographics, (3) neighbourhood characteristics, and (4) general practice characteristics. Regarding the health and healthcare use factors, we included the following independent variables for each of the included years: the number of (1) drug prescriptions from different Anatomical Therapeutic Chemical (ATC) main drug classes, (2) disease episodes, (3) acute symptoms, (4) long-lasting symptoms, (5) chronic diseases, (6) contacts with the general practice, (7) referrals to different specialities, and (8) primary care OOH contacts. Drugs were recorded using the ATC classification system, on the 3rd level, and were classified into 14 different main ATC classes (1st level, anatomical main group). Disease episodes were constructed from International Classification of Primary Care (ICPC) codes (version 1), which were recorded for each consultation [35]. Diagnoses were subsequently grouped into the following episode categories of disease: acute symptoms, long-lasting symptoms, and chronic diseases, using the methods described by Nielen et al. [36]. Primary care OOH contacts could be derived from the linked primary care OOH services dataset pertaining to OOH services consultations as described previously. Individual health and healthcare utilization data were limited to data routinely recorded by each individual’s general practice and/or primary care OOH service, but may also include data originating from other healthcare providers outside these settings, for both individuals with and without consent for electronic HIE (see Background section). The categorization of all health and healthcare use factors is presented in Table 2.

The following demographic characteristics were included to investigate differences in consent: (1) gender and (2) age. Age was categorized in nine categories: 0–11 years, 12–15 years, 16–24 years, 25–34 years, 35–44 years, 45–54 years, 55–64 years, 65–74 years and 75 years and older. The first three age categories were deliberately chosen, as children under 12 years are not legally allowed to provide consent for electronic HIE themselves. Children between 12 and 15 years can provide consent themselves but only if approved by their parent(s) or guardian(s). Individuals aged ≥ 16 years can provide consent themselves, independently from their parent(s) or guardian(s). Intervals of 10 years were chosen from 25 until 74 years; the last age group was categorized as 75 years and older to make sure that the oldest age category still consisted of sufficient persons, in relation to the size of the other age groups.

The following neighbourhood characteristics were included: (1) neighbourhood socioeconomic position (SEP), (2) degree of urbanization and (3) postcode (4-digit) area deprivation status of the individual’s area of residence. Neighbourhood SEP is defined as a combined score on the basis of welfare, education level and labour market participation of people living in a neighbourhood, and is centred around the value “0” for the whole Dutch population [37]. Neighbourhood SEP scores in each of the included years were based on the neighbourhood SEP score in 2017. As this score was not normally distributed, SEP scores were categorized into five evenly distributed neighbourhood SEP categories. The degree of urbanization variable was already categorized in the dataset, following the five categories of Statistics Netherlands [38]. The categorization of all neighbourhood characteristics can be found in Table 2.

The following practice characteristics were included: (1) type of EHR system, (2) practice size and (3) type of practice. Regarding the type of EHR system, there were two EHR systems included: type A and type B. During the study period, 10 general practice EHR systems existed, but most practices participating in Nivel-PCD used 1 of these 2 systems. Because the number of patients per general practice was not normally distributed, we categorized practice size into tertiles on the basis of the number of patients per general practice. The type of practice was categorized into three groups: single-handed, duo or group practices. Single-handed practices consist of one general practice owner, duo practices consist of two general practice owners and group practices consist of three or more general practice owners. The categorization of all practice characteristics is presented in Table 1.

**Table 1:** Baseline characteristics practices

Characteristic	2017 All practices (*n* = 46)	2018 All practices (*n* = 42)	2019 All practices (*n* = 65)
Practice type^*^, *n* (%)			
Single-handed	9 (19.6)	9 (21.4)	17 (26.2)
Duo	15 (32.6)	17 (40.5)	26 (40.0)
Group	22 (47.8)	16 (38.1)	22 (33.9)
EHR system, *n* (%)			
A	37 (80.4)	35 (83.3)	50 (76.9)
B	9 (19.6)	7 (16.7)	15 (23.1)
Practice size^**^, *n* (%)			
Small	16 (34.8)	14 (33.3)	22 (33.9)
Medium	15 (32.6)	14 (33.3)	22 (33.9)
Large	25 (32.6)	14 (33.3)	21 (32.3)

^*^Single-handed practices consist of one general practice owner; duo practices consist of two general practice owners; and group practices consist of three or more general practice owners. This categorization is independent of the number of other general practice staff working within the practice

^**^Because the number of patients per general practice was not normally distributed, we categorized practice size into tertiles on the basis of the number of patients per general practice

### Inclusion and exclusion criteria

For each of the included years from 2017 to 2019, we first checked the Nivel-PCD general practice EHR data and excluded individuals who were not registered with the same Nivel-PCD general practice for the entire year. Because (almost) every citizen in the Netherlands is registered with a GP, and every general practice is linked to a specific OOH service on the basis of its location, it was possible to link the pseudonymised data from each practice to a specific OOH service and take their OOH visits into account. In our analyses we included only individuals listed in practices in regions in which the OOH service was also participating in Nivel-PCD.

General practices with ≥ 1 individuals who had missing data regarding their consent registration were excluded, thereby preventing results from being affected by differences in the proportion of people for whom consent was (not) recorded, ensuring a comparable sample of general practices. Both in 2017 and 2018, this resulted in the exclusion of 50 general practices, of which the majority had missing consent registrations for at least a quarter of their patient population. Furthermore, regarding the practice type, relatively many group practices were excluded when compared with single-handed or duo practices. In 2019, a larger share of general practices had missing consent data (155 general practices). However, because the Nivel-PCD general practice EHR database from 2019 consisted of more general practices, a larger number of general practices still remained after this exclusion compared with 2017 and 2018. Regarding the general practice characteristics, all practices using two specific types of EHR systems were thereby excluded. Furthermore, relatively more duo practices were excluded when compared with single-handed or group practices. Finally, in preparation for the multilevel logistic regression analyses, we also excluded individuals with missing data for one or more of the included independent variables (see Additional File IV: Figs. 1–3, indicating the flowchart for determining the study sample selection). Persons in our final study databases for 2017, 2018 and 2019 were representative of the Dutch population regarding gender and age. However, the dispersion of individuals among urbanized or rural areas was not representative in our sample. General practices were not representative regarding practice type (i.e. single-handed, duo or group practice) (see Additional file II: Tables 1, 2).

### Hypotheses

Regarding the abovementioned measures, we explored which groups of individuals are more likely to provide consent for electronic HIE. Earlier studies from other countries showed that a patient’s decision concerning this consent is not exclusively dependent upon individual patient-level factors, but that the social context and policy context are also important to consider, and whether they perceive healthcare benefits [8, 20–32, 39]. Regarding health and healthcare utilization, we expected that individuals with higher healthcare needs (e.g. those with more prescriptions from different ATC main drug classes, frequent disease episodes, or more GP/primary care OOH contacts) are more likely to provide consent for electronic HIE. This is because they are expected to benefit more from electronic HIE between their (multiple) healthcare providers. However, because more medical data are available on patients with higher healthcare utilization, these patients may perceive a greater risk in the event of data leakage and, therefore, may be less inclined to provide consent for HIE. The organization that is nationally responsible for the National Exchange Point (i.e. VZVZ) engaged in successful campaigns to promote consent among vulnerable groups, who generally have higher healthcare needs and utilization and therefore more often visit their GP [40]. On the basis of such health policy context, we would still expect that these groups are potentially more likely to encounter the opportunity to provide consent for electronic HIE.

With regard to the patient demographic characteristics, we consequently also hypothesized that patients who generally have higher healthcare use in the Netherlands (i.e. women and elderly) are more likely to provide consent [41, 42]. In relation to the included neighbourhood factors, we therefore also expected that patients living in deprived areas and stronger urbanized areas are more likely to provide consent, as their healthcare utilization is generally higher [43, 44].

Finally, regarding the practice characteristics, we expected that patients who are registered in larger general practices (i.e. group practices and larger practice sizes) are more likely to provide consent as these practices have generally more resources to better inform their patients about the benefits of electronic HIE. Furthermore, we expected differences in consent between patients from practices with different EHR systems as earlier research indicated that healthcare provider’s satisfaction with their EHR, or EHR system, plays an important role in their ability to exchange health information electronically [45].

### Data analysis

Data analysis was performed using Stata (version 16.1; StataCorp LLC). Descriptive statistics were used to describe the outcome variable and the included individual-level and practice-level factors. To assess the association between these covariates and the outcome measure, separately for each of the included years, a bivariate chi-squared test was executed first, and afterwards, a multilevel logistic regression analysis was performed, with random effects at the practice level. First, a null-model with only the dependent variable was constructed to investigate the a priori chance of an individual providing consent for electronic HIE. In model 1, all health and healthcare utilization factors were added. In model 2, in addition, the demographic factors were included. In model 3, the neighbourhood factors were added. Finally, in model 4, the practice characteristics were included. For each of these covariates, the first category was chosen as reference group. The risk of confounding was minimized by including these factors simultaneously, thereby mutually adjusting for all independent variables. To establish the best “fit” of these models for each year, a log likelihood-ratio test was performed. Further, odds ratios (ORs), including the 95% confidence intervals, corresponding *P*-values and intraclass correlation coefficients (ICC) were calculated.

## Results

### Baseline characteristics

In total, individual-level and practice-level characteristics, and citizen consent for electronic general practice HIE data was available for 167 380–263 059 individuals from 42 to 65 general practices in the period between 2017 and 2019 (Tables 1 and 2). In all 3 years, the proportion of females and males was similar, and slightly more than half of the study population was aged 45 years or older. Further, a majority of the individuals had ≥ 1 chronic diseases (range: 59.3–62.0%). In addition, the largest share of citizens was registered in a group practice (range: 48.8–63.3%).

### Individual consent and practice variation

In 2017, 44.7% of the individuals explicitly provided consent for electronic general practice HIE (range: 1.3–97.3%; median: 37.3%, on practice level). In 2018 and 2019, this share was 38.4% (range: 1.3–95.7%; median: 27.9%, on practice level) and 44.0% (range: 1.1–100%; median: 42.8%, on practice level), respectively (Tables 1 and 2). These ranges indicate a very large practice variation in citizen consent in all 3 years. Overall, there was a reasonably high degree of overlap among the included practices across the 3 years studied (range: 61–75%; see Additional File I: Tables 1 and 2).

The multilevel logistic regression results from 2019 show that the chance that an individual provided consent for electronic HIE was 25% lower than the chance that an individual did not provide consent, in case the covariates are not considered and when adjusted for practice level clustering (i.e. a priori chance; Table 4; OR 0.75). In 2017 and 2018, these a priori chances were 0.54 and 0.38, respectively (Additional File III: Tables 1 and 2). In all 3 years, practice variance was high, meaning that differences between general practices played an important role in the study’s outcome. In 2019, for example, 56% of the variance in providing consent could be explained by differences between practices (Table 4; ICC 0.56, 95% confidence interval (CI) 0.47–0.65). In 2017 and 2018, about a quarter of the variance in providing consent could be explained by differences between practices (Additional File III: Tables 1 and 2; ICC 0.30 and 0.27, respectively).

### Factors related to providing consent for electronic HIE

Several factors were associated with providing consent for electronic HIE. The bivariate chi-squared test results show a significant association between citizen consent and independent study characteristics across all 3 analysed years. A larger share of the following groups provided consent for electronic HIE compared with their counterparts: individuals without health complaints or healthcare use (i.e. no main drug group prescriptions, no disease episodes, no acute/long-lasting/chronic diseases, and no GP/OOH contacts or referrals to specialists), men, younger age groups, individuals from practices using EHR system type B, and those from large-sized practices (Table 3).

The multilevel logistic regression analysis show partially similar results compared with these bivariate test results. With regard to the health and healthcare utilization factors (see model 4 in Table 4, Additional File III: Tables 1 and 2), in each of the 3 analysed years, a higher number of prescriptions from different ATC main drug classes per patient, presence of ≥ 1 long-lasting symptoms, or presence of ≥ 1 chronic diseases was significantly associated with lower odds of providing consent for electronic HIE (OR ≤ 0.94; *P* < 0.001). With reference to the demographic factors, for each of the included years, we found that females or individuals aged 65 years and older had lower odds of providing consent (OR ≤ 0.96; *P* ≤ 0.05), when corrected for the health and healthcare utilization factors. Regarding the neighbourhood factors, in all 3 years we found that citizens in below-average SEP neighbourhoods had higher odds of providing consent for electronic HIE compared with people with the lowest SEP (OR ≥ 1.09; *P* < 0.001), when corrected for the health and healthcare utilization factors and the demographic factors. Only in 2017, citizens with an average SEP also had higher odds for providing consent (OR 1.10, 95% CI 1.05–1.15). For 2 of the included years (i.e. 2017 and 2019), it was found that citizens living in hardly urbanized areas or in deprived (postal code) areas had higher odds of providing consent for electronic HIE (OR ≥ 1.05; *P* ≤ 0.05).

Further, only in 2017 did we find that patients with ≥ 2 different referrals had higher odds of providing consent compared with individuals without referrals (OR 1.06, 95% CI 1.00–1.11). Only in 2018 did we find that patients with ≥ 3 GP contacts or people aged 16–24 years had higher odds of providing consent compared with individuals without GP contact or people aged ≤ 11 years, respectively (OR ≥ 1.05; *P* < 0.05). However, individuals with ≥ 3 disease episodes, aged 12–15 years or 55–64 years or living in a moderately urbanized area had lower odds in 2018 of providing consent compared with citizens without disease episodes, aged ≤ 11 years, or living an extremely urbanized area, respectively (OR ≤ 0.95; *P* < 0.05). Further, patients with ≥ 1 GP contacts or patients having acute symptoms had lower odds of providing consent compared with people without GP contacts or not having acute symptoms in 2019, respectively (OR ≤ 0.93; *P* < 0.001).

Finally, in 2 of the included years, no significant differences were found with reference to the neighbourhood SEP of the individual, the presence of acute symptoms (both in 2017 and 2018) or the number of disease episodes (both in 2017 and 2019). In addition, in each of the 3 analysed years, no significant differences were found regarding the number of primary care OOH contacts and two of the included practice characteristics (i.e. EHR system and practice type) in relation to providing consent for electronic HIE. In 2017, patients from large-sized general practices had higher odds of providing consent compared with small-sized practices (OR 3.22, 95% CI 1.06–9.81), while in 2019, patients from medium-sized general practices had lower odds of providing consent compared with small-sized practices (OR 0.22, 95% CI 0.06–0.85). However, for both years we found that model 4, which included the practice characteristics, had a poorer fit compared with model 3 (i.e. with all individual-level factors but without practice characteristics). Because we were also interested in the differences in an individual’s consent for HIE in relation to the practice-level characteristics, and as the results of model 4 in 2017 and 2019 did not differ much from the model 3 results, and because model 4 had the best fit of all analysed models in 2018, we chose to report the model 4 results for each of the analysed years. Furthermore, the practice-level results of the multilevel analysis were already corrected for the health and healthcare utilization factors, demographic factors and neighbourhood factors. In all 3 years, we observed that individual-level characteristics could not explain the practice-level variation, as there was no change in ICC after including these characteristics. These differences could only hardly be explained by the included practice-level factors, as ICC only showed a minor decrease after including these factors.

## Discussion

### Main findings

This study, using routinely recorded EHR data from general practices in the Netherlands, shows that among practices that had been enrolled in the National Exchange Point, a minority of registered patients (38–45%) explicitly provided consent for electronic HIE at their general practice, in the period from 2017 to 2019. The remarkably high practice variation in all 3 years, that largely remained after correction for the included individual-level and practice-level factors, suggests that general practices play an important role in an individual’s choice to provide consent. Furthermore, we found that individuals with generally a higher healthcare utilization, who are expected to benefit most from the national HIE system, are less likely to provide consent, which is contrary to what we expected. In addition, the multilevel analysis showed that individuals, for whom patient information may be exchanged among healthcare providers from different organizations (i.e. in case of referrals to secondary care and in case of primary care OOH service contacts) were as likely to provide consent for electronic HIE in comparison with individuals without referrals or OOH service contacts. Furthermore, two of the three included practice characteristics (i.e. EHR system and practice type) were not associated with an individual’s choice to provide consent for electronic HIE, which is not in line with our prior expectations either.

### Discussion of hypotheses and comparison with existing literature

In contrast to what we hypothesized, in all 3 years we saw that individuals with a higher number of prescriptions from different ATC main drug classes, ≥ 1 long-lasting symptom(s), or ≥ 1 chronic disease(s), which are three covariates within the health and healthcare use category, were less likely to provide consent for electronic HIE compared with individuals with no drug use, no long-lasting symptoms, or no chronic diseases, respectively. In all 3 years, these associations were highly statistically significant, with ORs consistently deviating substantially from 1. Consequently, it seems that people with the highest healthcare need, who should benefit most from the possibility of HIE, are less likely to provide consent compared with persons with no or a lower healthcare need. Several other studies, performed in other countries (mainly in the United States but also in New Zealand, Canada, South Korea, China, the United Kingdom, and several other European countries), and published before the end of 2020, show that a patient’s intention to share data varies depending on the type of data recipient. In addition, these studies show that concerns and issues related to trust, identity, privacy and security are important factors that are considered when choosing to provide consent for electronic HIE [20, 46]. Esmaeilzadeh et al. [20], for example, states that patients with chronic diseases may be more concerned about their health data being shared through HIE [20]. It is possible that these factors (i.e. privacy and security) are also important factors for patients in the Netherlands with higher healthcare utilization when deciding whether to provide consent for electronic HIE. These concerns are less relevant for individuals with low healthcare needs or utilization, as they have little to no health data to be exchanged between healthcare providers. In short, willingness to consent can be seen as a pre-behavioural intention shaped by weighing benefits and risks; for example, patients with higher healthcare utilization may benefit more from data sharing owing to multiple providers involved in their care, yet also perceive greater risks to potential privacy and security issues, possibly reducing their willingness to consent. According to a systematic review by Cascini et al. [21], addressing these risks and concerns and spreading awareness about the benefits of health data sharing may lead to higher consent rates among the various patient groups [21]. Furthermore, Kassam et al. [47] showed in a literature review (including studies published in the period from 2010 to 2022, mainly from the United States and several European countries) that transparency on how data are used and having oversight mechanisms in place are also important for individuals to provide consent for sharing their general practice health data electronically [47]. As mentioned before, there are several exceptions to the national electronic opt-in system in the Netherlands, referred to as “push” HIE. In these cases, explicit consent from the patient is not required for the exchange of patient health data if the patient’s physician deems it medically necessary [16]. It is possible that this has also influenced the choice of individuals to provide consent for electronic HIE. As this effect is difficult to investigate and quantify, this highlights the importance of further (qualitative) research to explore the reasons for not providing explicit consent for electronic HIE among individuals in the Netherlands with higher healthcare needs or utilization.

Furthermore, regarding the demographic factors, we found that females and persons aged ≥ 65 years also had less of a chance of providing consent compared with the reference groups, i.e. males or persons aged ≤ 11 years, respectively, when corrected for health and healthcare utilization factors. Especially in 2018 and 2019, these associations were highly statistically significant. These results also contradict with our hypotheses. Earlier studies report miscellaneous results regarding the role of these demographic factors in predicting the willingness to share personal health data, which makes it difficult to draw firm conclusions. Regarding age, for example, other (survey) studies that were performed in the United States and Germany also found that older age was associated with a lower intention to share health data for primary use; however, no differences between males and females were observed [48–50]. However, another study by O’Donnell et al. [51], performed in the United States, showed that people interested in using personal HIE were less likely to be female, but found no differences between people aged under 65 years and those aged 65 years and older, while another study in the United States by Yaraghi [52] found that female patients were more likely to provide consent and the likelihood of providing consent increased by age [51, 52]. According to Dimotropoulos et al. [53], concerns regarding the security of HIE are significantly higher among people between the ages of 40 and 64 years compared with people between the ages of 18 and 39 years, which might explain why they are more reluctant to provide consent for HIE [53].

Regarding the neighbourhood factors, we observed that citizens from below-average SEP neighbourhoods and living in deprived areas usually had higher odds of providing consent for electronic HIE compared with the reference groups (i.e. lowest SEP and not deprived area, respectively). The association for individuals from below-average SEP was highly statistically significant in all 3 years, while significance was generally weaker for the association for people living in deprived areas. Earlier studies, however, found that patients with a relatively high income and patients with higher education levels are more likely to share their health information, which contradicts with our observations [20, 51, 54]. Patients living in hardly urbanized areas also had higher odds of providing consent, in 2 of the studied years. These results regarding neighbourhood factors were, however, generally weaker compared with the abovementioned results regarding the health and health use factors.

Furthermore, since the ICC did not change after including individual characteristics, and only slightly decreased in the multilevel analysis after adding the practice characteristics for each year, practice variation does not appear to be explained by individual characteristics and is only partly explained by practice characteristics. This suggests that unmeasured factors play a role. The ICC was relatively high in 2019, indicating that much of the variance in providing consent can be explained by differences between practices. However, it is likely that other practice characteristics than the practice type, practice size, and health information system, such as practice staff characteristics (e.g. beliefs about electronic HIE and previous experiences with HIE), practice composition (e.g. young or older population, (non)digitally skilled population) or practice communication strategies (e.g. face-to-face or digital), can further clarify this variance. The higher ICC value in 2019 might be related to the higher number of included practices compared with 2017 and 2018.

### Strengths and limitations

To the best of our knowledge, this study is the first to assess differences in individuals’ actual behaviour regarding consent for HIE, considering individual-level and practice-level factors in the general population, using routinely recorded data from general practices and primary care OOH services over several years. The large number of subjects contributed to a robust analysis and the possibility to systematically identify groups that are more likely to provide consent for electronic HIE. Furthermore, the use of EHR data is not dependent on subjective interpretation from individuals or primary care providers, making this data reliable. Consequently, these study results can adequately support policymakers to target the patient populations lagging behind, as these groups could benefit from better communication about the (dis)advantages of providing consent for electronic HIE.

This study also has limitations. We chose to depend on the availability and the fitness for purpose of routinely recorded data that were presumed to be relevant for the research question. Potentially relevant practice-level factors, including GP staff characteristics (e.g. age, gender, belief about electronic HIE and active promotion of electronic HIE benefits), could not be taken into account, as these were not available through the Nivel-PCD [32]. In addition, other than the included individual-level characteristics, such as education level, technology experience, HIE familiarity and an individual’s trust in healthcare providers, other factors might also be considered as important covariates for future research [49, 55].

In the final dataset, there were only general practices included from 2 (common) EHR systems, out of the approximately 10 EHR systems that existed in the Netherlands during that period. This means that it is possible that our results cannot be fully translated to individuals from general practices using other EHR systems. For future research, it would also be interesting to include general practices using other EHR systems to make more robust conclusions about their impact in relation to an individual’s decision to provide consent for electronic HIE.

Owing to the inclusion of multiple potentially relevant covariates in our multilevel analyses, some general practices and individuals were excluded from the primary database of our study. Specifically, this was because we included data on the number of referrals to different medical specialities and contacts with primary care OOH services. Regarding the referral data, information was unavailable for a relatively large share of practices. In addition, because the primary care OOH service contact data partially overlapped with the Nivel-PCD database from general practices, this resulted in missing consent values for a proportion of individuals. Nevertheless, we believe that the inclusion of these two covariates was important, as it allowed us to capture information on visits to primary care OOH services and referrals to specialist care. This provided valuable insights into consent differences between individuals who only visited daytime general practices and those who also accessed primary care OOH services or secondary care, for whom HIE is expected to be particularly beneficial. Furthermore, the final study populations were still representative regarding age and gender.

### Implications for research and practice

As we see that patients with multiple different drug prescriptions or older persons were less likely to provide HIE consent, we think it is important for policymakers and other related stakeholders to better communicate the benefits of this system, especially for these groups of citizens. This also encompasses improved communication with healthcare providers and educating them on this topic, as was also carried out in a successful pilot by VZVZ in 2021 [40]. In addition, it is also important to sufficiently inform and involve healthcare providers and discuss with them why they think these individuals are less likely to provide consent and which barriers they perceive (e.g. lack of trust in the national HIE system). Consequently, general practice staff may be able to better inform their patients, and especially the patients who are generally less easy to reach, about the (dis)advantages of electronic HIE, enabling their patients to make a well-informed decision about the use of this system, generally leading to higher support of HIE initiatives. In addition, supporting patients with the use of technology and improving their digital skills is also thought to positively impact patient perceptions about HIE [20]. With regard to age, however, as the population ages, differences in consent rates between younger and older populations may naturally diminish over time, suggesting that there may be no urgent need to specifically target older individuals in this regard.

Qualitative research could also be useful to negate the reasons why specific groups of individuals are more or less likely to provide consent for electronic HIE. This then helps to involve citizens and healthcare providers in the further exploitation of the national HIE infrastructure. Further research could also focus on what the health consequences are for individuals when they do not provide consent, and how this affects healthcare providers’ work and ability to provide good care to these groups in comparison with people who provided consent for HIE.

With the development and implementation of the EHDS, which should enable safe and secure exchange of an individual’s health data among healthcare providers of European Member States, it is also important to investigate citizens’ and healthcare providers’ view on this subject at an early stage and involve them during this process of implementation. Although a European political agreement has been reached on the implementation of an opt-out system for the exchange of health data, which differs from the current national electronic HIE system in the Netherlands, there is also room for Member State-specific legislation for specific types of data [17]. Insights from our study and insights from other European Member States about this subject are therefore important to take into consideration and may support rethinking the organization of the national HIE infrastructure, which may impact citizen consent rates [8, 13, 14]. In addition, the changing relationships between patients and healthcare providers (e.g. patient empowerment) may also be a reason to rethink the organization of this infrastructure, as patients also increasingly manage their own health data [56, 57].

Finally, future research could also focus on how consent rates in the Netherlands evolved between different groups of individuals after the COVID-19 pandemic. The temporary COVID-19 regulation, which affected consent registration, stopped in April 2023. Post-COVID, it is relevant to investigate differences in consent rates between patients with specific diseases (e.g. mental disorders versus physical complaints), as for some disorders or illnesses people might be more hesitant to share their health data electronically [46].

## Conclusions

Between 2017 and 2019, less than half of the population, listed as patients in practices in which electronic HIE was possible, provided explicit consent for electronic general practice HIE. Individuals with higher healthcare utilization were generally less likely to provide consent. Individuals from below-average SEP neighbourhoods and living in deprived urban or hardly urbanized regions usually were more likely to provide consent. These findings suggests that the current opt-in system in the Netherlands may not adequately serve the majority of people who could benefit most from this electronic HIE system. As a relatively large share of the variance in providing consent can be attributed to differences between general practices, involvement of GPs in electronic HIE system improvements, enhancing transparency, and their role in addressing barriers perceived by individuals are considered to be crucial.

## Supplementary Information

Additional file 1. I. Overview of included study practices, 2017-2019. Description of data: Overview of the number, and similarity between included study general practices in each of the measurement years (Addition file I: Table 1 and Table 2). II. Representativeness of the final datasets, 2017-2019. Description of data: Overview of the representativeness of the study population (general practices and citizens), based on national figures on the distribution of general practice type, and the distribution of gender, age category, and degree of urbanization of the general population (Additional file II: Table 1 and Table 2). III. Results multilevel logistic regression analyses, 2017 and 2018. Description of data: Results of the multilevel logistic regression analyses regarding citizen choice to provide consent for electronic HIE in 2017 and 2018 (Additional file III: Table 1 and Table 2). IV. Flowchart for study sample selection, 2017-2019. Description of data: Overview of the inclusion and exclusion criteria for determining the study population (Addition file IV: Table 1 and Table 2).

## Data Availability

Access to the data underlying this article can be facilitated after obtaining approval from the steering committees of the Nivel Primary Care Database. It is important to note that the dataset is not freely available; permission must be sought, along with potential associated fees, before access is granted. These arrangements are in place to ensure proper data usage (https://www.nivel.nl/en/our-databases-and-panels/nivel-primary-care-database). For more information about the dataset’s specifics as used in this study, enquiries can be directed to the corresponding author.

## Appendix

See Table 2, 3, and 4.

**Table 2:** Baseline characteristics of citizens

Characteristic	2017 All citizens (*n* = 175 230)	2018 All citizens (*n* = 167 380)	2019 All citizens (*n* = 263 059)
Citizen consent, *n* (%)			
Yes	78 284 (44.7)	64 227 (38.4)	115 862 (44.0)
No	96 946 (55.3)	103 153 (61.6)	147 197 (56.0)
Health and healthcare use			
No. of different main drug group prescriptions, *n* (%)			
0	49 761 (28.4)	47 683 (28.5)	74 339 (28.3)
1	37 379 (21.3)	36 107 (21.6)	56 550 (21.5)
2 or 3	48 023 (27.4)	45 564 (27.2)	71 901 (27.3)
≥ 4	40 067 (22.9)	38 026 (22.7)	60 269 (22.9)
No. of disease episodes, *n* (%)			
0	17 180 (9.8)	15 622 (9.3)	23 697 (9.0)
1 or 2	44 708 (25.5)	41 980 (25.1)	64 657 (24.6)
≥ 3	113 342 (64.7)	109 778 (65.6)	174 705 (66.4)
No. of acute symptoms, *n* (%)			
0	45 163 (25.8)	42 250 (25.2)	66 779 (25.4)
≥ 1	130 067 (74.2)	125 130 (74.8)	196 280 (74.6)
No. of long-lasting symptoms, *n* (%)			
0	72 694 (41.5)	68 008 (40.6)	105 309 (40.0)
≥ 1	102 536 (58.5)	99 372 (59.4)	157 750 (60.0)
No. of chronic diseases, *n* (%)			
0	71 404 (40.8)	66 162 (39.5)	99 944 (38.0)
≥ 1	103 826 (59.3)	101 218 (60.5)	163 115 (62.0)
No. of GP contacts, *n* (%)			
0	38 047 (21.7)	35 714 (21.3)	55 560 (21.1)
1–2	49 062 (28.0)	46 994 (28.1)	71 566 (27.2)
≥ 3	88 121 (50.3)	84 672 (50.6)	135 933 (51.7)
No. of referrals to different medical specialities, *n* (%)			
0	133 673 (76.3)	128 040 (76.5)	199 011 (75.7)
1	32 837 (18.7)	31 127 (18.6)	50 011 (19.0)
≥ 2	8720 (5.0)	8213 (4.9)	14 037 (5.3)
No. of OOH contacts, *n* (%)			
0	149 559 (85.4)	149 937 (89.6)	224 491 (85.3)
1	18 762 (10.7)	12 887 (7.7)	28 452 (10.8)
≥ 2	6909 (3.9)	4556 (2.7)	10 116 (3.9)
Demographics			
Gender, *n* (%)			
Male	86 915 (49.6)	83 129 (49.7)	130 359 (49.6)
Female	88 315 (50.4)	84 251 (50.3)	132 700 (50.4)
Age category (years), *n* (%)			
≤ 11	19 169 (10.9)	18 694 (11.2)	29 069 (11.1)
12–15	8505 (4.9)	8078 (4.8)	12 589 (4.8)
16–24	19 230 (11.0)	18 156 (10.8)	28 595 (10.9)
25–34	18 865 (10.8)	18 103 (10.8)	28 471 (10.8)
35–44	19 390 (11.1)	18 874 (11.3)	30 123 (11.5)
45–54	27 476 (15.7)	25 269 (15.1)	38 022 (14.5)
55–64	25 502 (14.6)	24 396 (14.6)	38 901 (14.8)
65–74	20 877 (11.9)	20 504 (12.3)	33 421 (12.7)
≥ 75	16 216 (9.3)	15 306 (9.1)	23 868 (9.1)
Neighbourhood characteristics			
Neighbourhood SEP, *n* (%)			
Lowest	37 861 (21.6)	35 143 (21.0)	53 120 (20.2)
Below average	32 412 (18.5)	35 786 (21.4)	52 415 (19.9)
Average	34 895 (19.9)	29 861 (17.8)	52 738 (20.1)
Above average	36 857 (21.0)	33 152 (19.8)	57 045 (21.7)
Highest	33 205 (19.0)	33 438 (20.0)	47 741 (18.2)
Degree of urbanization, *n* (%)			
Extremely	18 540 (10.6)	16 099 (9.6)	41 375 (15.7)
Strongly	31 946 (18.2)	29 168 (17.4)	65 345 (24.8)
Moderately	29 321 (16.7)	34 191 (20.4)	69 347 (26.4)
Hardly	49 027 (28.0)	45 821 (27.4)	43 870 (16.7)
Not	46 396 (26.5)	42 101 (25.2)	43 122 (16.4)
Area deprivation status (postal code), *n* (%)			
Not deprived	171 207 (97.7)	163 821 (97.9)	245 339 (93.3)
Deprived	4023 (2.3)	3559 (2.1)	17 720 (6.7)
Practice-level characteristics			
Practice type, *n* (%)			
Single-handed	22 073 (12.6)	31 602 (18.9)	55 655 (21.2)
Duo	42 182 (24.1)	50 233 (30.0)	79 154 (30.1)
Group	110 975 (63.3)	85 545 (51.1)	128 250 (48.8)
EHR system, *n* (%)			
A	127 594 (72.8)	132 673 (79.3)	194 673 (74.0)
B	47 636 (27.2)	34 707 (20.7)	68 386 (26.0)
Practice size, *n* (%)			
Small	37 592 (21.5)	32 210 (19.2)	49 560 (18.8)
Medium	42 682 (24.4)	40 758 (24.4)	65 498 (24.9)
Large	94 956 (54.2)	94 412 (56.4)	148 001 (56.3)

**Table 3:** Bivariate chi-squared test results for the association between citizen consent and independent study characteristics, 2017–2019

Characteristic	2017 All citizens (*n* = 175 230)		2018 All citizens (*n* = 167 380)		2019 All citizens (*n* = 263 059)	
	Citizens providing consent	*P*-value Pearson’s chi-squared test	Citizens providing consent	*P*-value Pearson’s chi-squared test	Citizens providing consent	*P*-value Pearson’s chi-squared test
Citizen consent, *n* (%)						
Yes	78 284 (44.7)		64 227 (38.4)		115 862 (44.0)	
No	96 946 (55.3)		103 153 (61.6)		147 197 (56.0)	
Health and healthcare use						
No. of different main drug group prescriptions, *n* (%)						
0	25 783 (51.8)	** < 0.001**	22 600 (47.4)	** < 0.001**	36 942 (49.7)	** < 0.001**
1	17 521 (46.9)		14 743 (40.8)		25 613 (45.3)	
2 or 3	20 303 (42.3)		15 955 (35.0)		30 511 (42.4)	
≥ 4	14 677 (36.6)		10 929 (28.7)		22 796 (37.8)	
No. of disease episodes, *n* (%)						
0	9213 (53.6)	** < 0.001**	7863 (50.3)	** < 0.001**	12 570 (53.0)	** < 0.001**
1 or 2	22 131 (49.5)		18 742 (44.7)		31 141 (48.2)	
≥ 3	46 940 (41.4)		37 622 (34.3)		72 151 (41.3)	
No. of acute symptoms, *n* (%)						
0	22 174 (49.1)	** < 0.001**	18 658 (44.2)	** < 0.001**	32 830 (49.2)	** < 0.001**
≥ 1	56 110 (43.1)		45 569 (36.4)		83 032 (42.3)	
No. of long-lasting symptoms, *n* (%)						
0	35 899 (49.4)	** < 0.001**	30 131 (44.3)	** < 0.001**	50 369 (47.8)	** < 0.001**
≥ 1	42 385 (41.3)		34 096 (34.3)		65 493 (41.5)	
No. of chronic diseases, *n* (%)						
0	35 314 (49.5)	** < 0.001**	29 376 (44.4)	** < 0.001**	48 067 (48.1)	** < 0.001**
≥ 1	42 970 (41.4)		34 851 (34.4)		67 795 (41.6)	
No. of GP contacts, *n* (%)						
0	18 966 (49.9)	** < 0.001**	16 048 (44.9)	** < 0.001**	27 720 (49.9)	** < 0.001**
1–2	22 623 (46.1)		18 881 (40.2)		32 522 (45.4)	
≥ 3	36 695 (41.6)		29 298 (34.6)		55 620 (40.9)	
No. of referrals to different medical specialities, *n* (%)						
0	61 034 (45.7)	** < 0.001**	50 770 (39.7)	** < 0.001**	89 040 (44.7)	** < 0.001**
1	13 726 (41.8)		10 793 (34.7)		20 992 (42.0)	
≥ 2	3524 (40.4)		2664 (32.4)		5830 (41.5)	
No. of OOH contacts, *n* (%)						
0	67 509 (45.1)	** < 0.001**	59 378 (39.6)	** < 0.001**	99 289 (44.2)	** < 0.001**
1	7930 (42.3)		3649 (28.3)		12 254 (43.1)	
≥ 2	2845 (41.2)		1200 (26.3)		4319 (42.7)	
Demographics						
Gender, *n* (%)						
Male	40 194 (46.3)	** < 0.001**	33 640 (40.5)	** < 0.001**	58 799 (45.1)	** < 0.001**
Female	38 090 (43.1)		30 587 (36.3)		57 063 (43.0)	
Age category (years), *n* (%)						
≤ 11	9125 (47.6)	** < 0.001**	7948 (42.5)	** < 0.001**	13 944 (48.0)	** < 0.001**
12–15	4051 (47.6)		3410 (42.2)		5865 (46.6)	
16–24	9021 (46.9)		7703 (42.4)		13 446 (47.0)	
25–34	8833 (46.8)		7382 (40.8)		13 191 (46.3)	
35–44	8721 (45.0)		7295 (38.7)		13 593 (45.1)	
45–54	12 513 (45.5)		10 065 (39.8)		17 371 (45.7)	
55–64	11 176 (43.8)		9132 (37.4)		17 100 (44.0)	
65–74	8499 (40.7)		6545 (31.9)		12 748 (38.1)	
≥ 75	6345 (39.1)		4747 (31.0)		8604 (36.1)	
Neighbourhood characteristics						
Neighbourhood SEP, *n* (%)						
Lowest	14 716 (38.9)	** < 0.001**	12 908 (36.7)	** < 0.001**	27 420 (51.6)	** < 0.001**
Below average	14 157 (43.7)		14 109 (39.4)		28 909 (55.2)	
Average	18 489 (53.0)		12 730 (42.6)		19 083 (36.2)	
Above average	14 593 (39.6)		10 835 (32.7)		20 906 (36.7)	
Highest	16 329 (49.2)		13 645 (40.8)		19 544 (40.9)	
Degree of urbanization, *n* (%)						
Extremely	5989 (32.3)	** < 0.001**	3906 (24.3)	** < 0.001**	17 998 (43.5)	** < 0.001**
Strongly	16 363 (51.2)		13 231 (45.4)		24 976 (38.2)	
Moderately	12 371 (42.2)		11 590 (33.9)		31 684 (45.7)	
Hardly	24 354 (49.7)		19 825 (43.3)		19 185 (43.7)	
Not	19 207 (41.4)		15 675 (37.2)		22 019 (51.1)	
Area deprivation status (postal code), *n* (%)						
Not deprived	77 154 (45.1)	** < 0.001**	63 346 (38.7)	** < 0.001**	106 083 (43.2)	** < 0.001**
Deprived	1130 (28.1)		881 (24.8)		9779 (55.2)	
Practice-level characteristics						
Practice type, *n* (%)						
Single-handed	6870 (31.1)	** < 0.001**	10 052 (31.8)	** < 0.001**	28 040 (50.4)	** < 0.001**
Duo	14 216 (33.7)		12 063 (24.0)		29 833 (37.7)	
Group	57 198 (51.5)		42 112 (49.2)		57 989 (45.2)	
EHR system, *n* (%)						
A	52 196 (40.9)	** < 0.001**	45 062 (34.0)	** < 0.001**	81 765 (42.0)	** < 0.001**
B	26 088 (54.8)		19 165 (55.2)		34 097 (49.9)	
Practice size, *n* (%)						
Small	11 962 (31.8)	** < 0.001**	9533 (29.6)	** < 0.001**	23 811 (48.0)	** < 0.001**
Medium	12 899 (30.2)		9087 (22.3)		18 042 (27.6)	
Large	53 423 (56.3)		45 607 (48.3)		74 009 (50.0)	

*P*-values below 0.05 are considered statistically significant and are marked in bold

**Table 4:** Results of multilevel logistic regression analysis (2019), *n* = 263 059 citizens from 65 GP practices

	Null model		Model 1		Model 2		Model 3		Model 4	
	OR	95% CI	OR	95% CI	OR	95% CI	OR	95% CI	OR	95% CI
Overall outcome	0.75	0.44–1.30								
Health and healthcare use										
No. of different main group prescriptions										
0 (ref)			1.0		1.0		1.0		1.0	
1			**0.92** ^**a**^	**0.89–0.95**	**0.93** ^**a**^	**0.91–0.96**	**0.93** ^**a**^	**0.91–0.96**	**0.93** ^**a**^	**0.91–0.96**
2 or 3			**0.84** ^**a**^	**0.81–0.86**	**0.88** ^**a**^	**0.85–0.90**	**0.87** ^**a**^	**0.85–0.90**	**0.87** ^**a**^	**0.85–0.90**
≥ 4			**0.70** ^**a**^	**0.67–0.72**	**0.79** ^**a**^	**0.76–0.82**	**0.79** ^**a**^	**0.76–0.82**	**0.79** ^**a**^	**0.76–0.82**
No. of disease episodes										
0 (ref)			1.0		1.0		1.0		1.0	
1 or 2			1.02	0.98–1.07	1.00	0.96–1.05	1.00	0.96–1.05	1.00	0.96–1.05
≥ 3			0.99	0.93–1.05	0.98	0.92–1.04	0.98	0.93–1.04	0.98	0.93–1.04
No. of acute symptoms										
0 (ref)			1.0		1.0		1.0		1.0	
≥ 1			**0.94** ^**a**^	**0.91–0.97**	**0.93** ^**a**^	**0.90–0.97**	**0.93** ^**a**^	**0.90–0.96**	**0.93** ^**a**^	**0.90–0.96**
No. of long-lasting symptoms										
0 (ref)			1.0		1.0		1.0		1.0	
≥ 1			**0.93** ^**a**^	**0.91–0.95**	**0.93** ^**a**^	**0.90–0.96**	**0.93** ^**a**^	**0.91–0.96**	**0.93** ^**a**^	**0.91–0.96**
No. of chronic diseases										
0 (ref)			1.0		1.0		1.0		1.0	
≥ 1			**0.89** ^**a**^	**0.87–0.91**	**0.94** ^**a**^	**0.92–0.97**	**0.94** ^**a**^	**0.92–0.97**	**0.94** ^**a**^	**0.92–0.97**
No. of GP contacts										
0 (ref)			1.0		1.0		1.0		1.0	
1–2			**0.91** ^**a**^	**0.88–0.95**	**0.91** ^**a**^	**0.88–0.94**	**0.91** ^**a**^	**0.88–0.94**	**0.91** ^**a**^	**0.88–0.94**
≥ 3			**0.88** ^**a**^	**0.84–0.91**	**0.88** ^**a**^	**0.84–0.91**	**0.88** ^**a**^	**0.84–0.91**	**0.88** ^**a**^	**0.84–0.91**
No. of referrals to different specialisms										
0 (ref)			1.0		1.0		1.0		1.0	
1			0.98	0.96–1.01	0.99	0.97–1.02	0.99	0.97–1.02	0.99	0.97–1.02
≥ 2			**0.95** ^**c**^	**0.91–0.99**	0.96	0.92–1.01	0.96	0.92–1.01	0.96	0.92–1.01
No. of OOH contacts										
0 (ref)			1.0		1.0		1.0		1.0	
1			**1.03** ^**c**^	**1.00–1.07**	1.01	0.98–1.04	1.01	0.98–1.04	1.01	0.98–1.04
≥ 2			1.05	1.00–1.10	1.01	0.96–1.06	1.01	0.96–1.06	1.01	0.96–1.06
Demographics										
Gender										
Male (ref)					1.0		1.0		1.0	
Female					**0.96** ^**a**^	**0.94–0.97**	**0.96** ^**a**^	**0.94–0.97**	**0.96** ^**a**^	**0.94–0.97**
Age category (years)										
≤ 11 (ref)					1.0		1.0		1.0	
12–15					**0.91** ^**a**^	**0.86–0.95**	**0.91** ^**a**^	**0.86–0.95**	**0.91** ^**a**^	**0.86–0.95**
16–24					0.99	0.95–1.03	0.99	0.95–1.03	0.99	0.96–1.03
25–34					**0.93** ^**a**^	**0.89–0.97**	**0.93** ^**a**^	**0.89–0.97**	**0.93** ^**a**^	**0.89–0.97**
35–44					**0.90** ^**a**^	**0.86–0.93**	**0.90** ^**a**^	**0.86–0.93**	**0.90** ^**a**^	**0.86–0.93**
45–54					0.96	0.93–1.00	0.96	0.93–1.00	0.96	0.93–1.00
55–64					**0.90** ^**a**^	**0.86–0.93**	**0.90** ^**a**^	**0.86–0.93**	**0.90** ^**a**^	**0.86–0.93**
65–74					**0.71** ^**a**^	**0.68–0.74**	**0.71** ^**a**^	**0.68–0.74**	**0.71** ^**a**^	**0.68–0.74**
≥ 75					**0.68** ^**a**^	**0.65–0.72**	**0.69** ^**a**^	**0.65–0.72**	**0.68** ^**a**^	**0.65–0.72**
Neighbourhood characteristics										
Neighbourhood SEP										
Lowest (ref)							1.0		1.0	
Below average							**1.14** ^**a**^	**1.09–1.19**	**1.14** ^**a**^	**1.09–1.19**
Average							1.00	0.96–1.04	1.00	0.96–1.04
Above average							0.98	0.94–1.02	0.98	0.94–1.02
Highest							1.00	0.96–1.04	1.00	0.96–1.05
Degree of urbanization										
Extremely (ref)							1.0		1.0	
Strongly							0.99	0.96–1.03	0.99	0.96–1.03
Moderately							1.01	0.96–1.06	1.01	0.96–1.06
Hardly							**1.12** ^**a**^	**1.06–1.19**	**1.12** ^**a**^	**1.06–1.19**
Not							**1.09** ^**b**^	**1.03–1.16**	**1.09** ^**b**^	**1.03–1.16**
Area deprivation status (postal code)										
Not deprived (ref)							1.0		1.0	
Deprived							**1.05** ^**c**^	**1.00–1.09**	**1.05** ^**c**^	**1.00–1.09**
Practice-level characteristics										
EHR system										
A (ref)									1.0	
B									0.99	0.29–3.37
Practice type										
Single-handed (ref)									1.0	
Duo									0.84	0.21–3.28
Group									0.48	0.10–2.34
Practice size										
Small (ref)									1.0	
Medium									**0.22** ^**c**^	**0.06–0.85**
Large									1.28	0.29–5.64
Random effects										
Intercept var	4.91		4.95		4.96		4.91		4.23	
ICC	0.60		0.60		0.60		0.60		0.56	
Model fit										
Log likelihood	−140 495.88		−139 165.61		−138 863.24		−138 823.3		−138 818.75	
*N* citizens	263 059		263 059		263 059		263 059		263 059	
*N* practices	65		65		65		65		65	

^a^*P* < 0.001; ^b^*P* < 0.01; ^c^*P* < 0.05; ref, reference; var, variance

*P*-values below 0.05 are considered statistically significant and are marked in bold

## Abbreviations

ATC: Anatomical Therapeutic Chemical
EHR: Electronic health record
EHDS: European Health Data Space
GP: General practitioner
HIE: Health information exchange
ICC: Intraclass correlation coefficient
ICPC: International Classification of Primary Care
LSP: Landelijk Schakelpunt (National Exchange Point)
Nivel-PCD: Nivel Primary Care Database
OR: Odds ratio
OOH: Out-of-hours
US: United States
VZVZ: Vereniging van Zorgaanbieders voor Zorgcommunicatie (Association of Healthcare Providers for Healthcare Communication)
