# Correction: Determinants of consent for electronic health information exchange: an observational retrospective study

**DOI:** 10.1186/s12961-025-01391-z

**Published:** 2025-08-28

**Authors:** Jelle Keuper, Karin Hek, Lilian H. D. van Tuyl, Ronald Batenburg, Robert Verheij

**Affiliations:** 1 Netherlands Institute for Health Services Research, Utrecht, Netherlands; 2 Tranzo, Tilburg School of Social and Behavioral Sciences, Tilburg University, Tilburg, Netherlands; 3 Department of Sociology, Radboud University Nijmegen, Nijmegen, Netherlands; 4 National Health Care Institute, Diemen, Netherlands

**Correction: Health Research Policy and Systems (2025) 23:103** 10.1186/s12961-025-01377-x

Following publication of the original article [1], a typographical error in Table 2. In the rows for the Age categories for 25–34 and 35–44 under the column 2019 there was a decimal in the values.

The incorrect and correct section of Table 2 are given in this Correction with the corrected values in bold.

The original article has been updated.

**Incorrect Table 2 section**:

**Table:** 

Age category (years), *n* (%)			
≤ 11	19 169 (10.9)	18 694 (11.2)	29 069 (11.1)
12–15	8505 (4.9)	8078 (4.8)	12 589 (4.8)
16–24	19 230 (11.0)	18 156 (10.8)	28 595 (10.9)
25–34	18 865 (10.8)	18 103 (10.8)	28.471 (10.8)
35–44	19 390 (11.1)	18 874 (11.3)	30.123 (11.5)

**Correct Table 2 Section**

**Table:** 

Age category (years), *n* (%)			
≤ 11	19 169 (10.9)	18 694 (11.2)	29 069 (11.1)
12–15	8505 (4.9)	8078 (4.8)	12 589 (4.8)
16–24	19 230 (11.0)	18 156 (10.8)	28 595 (10.9)
25–34	18 865 (10.8)	18 103 (10.8)	**28 471** (10.8)
35–44	19 390 (11.1)	18 874 (11.3)	**30 123** (11.5)

## References

[1] Keuper J, Hek K, van Tuyl LHD, Batenburg R, Verheij R. Determinants of consent for electronic health information exchange: an observational retrospective study. Health Res Policy Syst. 2025;23:103. 10.1186/s12961-025-01377-x. 40775343 10.1186/s12961-025-01377-x PMC12333170

