# Supplementary material for: Determinants of consent for electronic health information exchange: an observational retrospective study
Source: Health Res Policy Syst. 2025 Aug 7;23:103. doi: 10.1186/s12961-025-01377-x (PMC12333170; doi:10.1186/s12961-025-01377-x)
Supplement: Supplementary file 1 — Additional file 1. I. Overview of included study practices, 2017-2019. Description of data: Overview of the number, and similarity between included study general practices in each of the measurement years (Addition file I: Table 1 and Table 2). II. Representativeness of the final datasets, 2017-2019. Description of data: Overview of the representativeness of the study population (general practices and citizens), based on national figures on the distribution of general practice type, and the distribution of gender, age category, and degree of urbanization of the general population (Additional file II: Table 1 and Table 2). III. Results multilevel logistic regression analyses, 2017 and 2018. Description of data: Results of the multilevel logistic regression analyses regarding citizen choice to provide consent for electronic HIE in 2017 and 2018 (Additional file III: Table 1 and Table 2). IV. Flowchart for study sample selection, 2017-2019. Description of data: Overview of the inclusion and exclusion criteria for determining the study population (Addition file IV: Table 1 and Table 2). [file 12961_2025_1377_MOESM1_ESM.docx]

## Additional file I – Overview of included study practice, 2017-2019

#### Addition file I : Table 1 – Number of practices with data for only 1 measurement year (2017-2019)

| **Year** | **Number of practice IDs having data from only 1 measurement year** |
| --- | --- |
| 2017 | 6 |
| 2018 | 1 |
| 2019 | 18 |

#### Additional file I: Table 2 – Similarity between study practice IDs (2017-2019)

| **Matching practice IDs between 2 or 3 years** | **Number of shared practice IDs** | **Percentage compared to mean number of shared practice IDs** | **Percentage compared to total number of unique practices (n=73)** |
| --- | --- | --- | --- |
| 2017 & 2018 | 33 | 33/44 = 75.0% | 33/73 = 45.2% |
| 2018 & 2019 | 40 | 40/53.5 = 74.8% | 40/73 = 54.8% |
| 2017 & 2019 | 38 | 38/55.5 = 68.5% | 38/73 = 52.1% |
| 2017, 2018 & 2019 | 31 | 31/51 = 60.8% | 31/73 = 42.5% |

## Additional file II – Representativeness of the final datasets, 2017-2019

#### Additional file II: Table 1 – Representativeness of study subjects, based on age, gender, and degree of urbanization (2017-2019)

| **Citizen characteristic** | **2017** (Study sample) | **2017** (the Netherlands)* | **2018** (Study sample) | **2018** (the Netherlands)* | **2019** (Study sample) | **2019** (the Netherlands)* |
| --- | --- | --- | --- | --- | --- | --- |
| **Age category**  ≤11  12-15  16-24  25-34  35-44  45-54  55-64  65-74  ≥75 | 10.9%  4.9%  11.0%  10.8%  11.1%  15.7%  14.6%  11.9%  9.3% | 12.1%  4.6%  11.1%  12.5%  12.0%  14.9%  13.4%  11.0%  8.4% | 11.2%  4.8%  10.9%  10.8%  11.3%  15.1%  14.6%  12.3%  9.1% | 12.0%  4.5%  11.1%  12.7%  11.9%  14.6%  13.5%  11.1%  8.6% | 11.1%  4.8%  10.9%  10.8%  11.5%  14.5%  14.8%  12.7%  9.1% | 11.9%  4.4%  11.1%  12.8%  11.9%  14.2%  13.7%  11.2%  8.9% |
| **Gender (female)** | 50.4% | 50,4% | 50.3% | 50.4% | 50.4% | 50.3% |
| **Degree of urbanization**  extremely  strongly  moderate  hardly  not | 10.6%  18.2%  16.7%  28.0%  26.5% | 23.1%  24.9%  17.3%  17.3%  17.3% | 9.6%  17.4%  20.4%  27.4%  25.2% | 23.3%  25.0%  17.2%  17.3%  17.1% | 15.7%  24.8%  26.4%  16.7%  16.4% | 23.6%  25.2%  17.1%  17.2%  17.0% |

*National-level figures are sourced from: [StatLine - Bevolking op 1 januari en gemiddeld; geslacht, leeftijd en regio](https://opendata.cbs.nl/#/CBS/nl/dataset/03759ned/table?dl=39E0B)

#### Additional file II: Table 2 – Representativeness of study practices, based on practice type (2017-2019)

| Practice characteristic | **2017** (Study sample) | **2017** (the Netherlands)* | **2018** (Study sample) | **2018** (the Netherlands)* | **2019** (Study sample) | **2019** (the Netherlands)* |
| --- | --- | --- | --- | --- | --- | --- |
| **Practice type, n(%)**  Single-handed  Duo  Group | 19.6%  32.6%  47.8% | 34.1%  42.5%  23.4% | 21.4%  40.5%  38.1% | 35.1%  41.5%  23.4% | 26.2%  40.0%  33.8% | 35.0%  41.8%  23.2% |

*National-level figures are sourced from: [Cijfers-uit-de-registratie-huisartsen-2017.pdf (nivel.nl)](https://www.nivel.nl/sites/default/files/pdf/Cijfers-uit-de-registratie-huisartsen-2017.pdf) (2017), [1003835.pdf (nivel.nl)](https://www.nivel.nl/sites/default/files/bestanden/1003835.pdf) (2018), [1004340.pdf (nivel.nl)](https://www.nivel.nl/sites/default/files/bestanden/1004340.pdf) (2019)

## Additional file III – Results multilevel logistic regression analyses, 2017 and 2018

#### Additional file III: Table 1 – Results multilevel logistic regression analysis (2017), n=174,936 citizens from 46 GP practices

|  | Null model | | Model 1 | | Model 2 | | Model 3 | | Model 4 | |
| --- | --- | --- | --- | --- | --- | --- | --- | --- | --- | --- |
|  | **OR** | **95% CI** | **OR** | **95% CI** | **OR** | **95% CI** | **OR** | **95% CI** | **OR** | **95% CI** |
| Overall outcome | **0.54^b^** | **0.37-0.80** |  |  |  |  |  |  |  |  |
| Health and healthcare use |  |  |  |  |  |  |  |  |  |  |
| # of different main group prescriptions  0 (ref)  1  2 or 3  ≥4 |  |  | 1.0  **0.82^a^**  **0.70^a^**  **0.54^a^** | **0.80-0.85**  **0.67-0.72**  **0.52-0.57** | 1.0  **0.83^a^**  **0.71^a^**  **0.56^a^** | **0.80-0.86**  **0.68-0.74**  **0.54-0.59** | 1.0  **0.83^a^**  **0.71^a^**  **0.56^a^** | **0.81-0.86**  **0.68-0.74**  **0.54-0.59** | 1.0  **0.83^a^**  **0.71^a^**  **0.56^a^** | **0.81-0.86**  **0.68-0.74**  **0.54-0.59** |
| # Disease episodes  0 (ref)  1 or 2  ≥3 |  |  | 1.0  1.02  0.98 | 0.96-1.07  0.92-1.05 | 1.0  1.01  0.98 | 0.96-1.07  0.92-1.06 | 1.0  1.01  0.98 | 0.96-1.07  0.92-1.05 | 1.0  1.01  0.98 | 0.96-1.07  0.92-1.05 |
| # Acute symptoms  0 (ref)  ≥1 |  |  | 1.0  1.01 | 0.97-1.05 | 1.0  1.01 | 0.97-1.05 | 1.0  1.01 | 0.97-1.05 | 1.0  1.01 | 0.97-1.05 |
| # Long-lasting symptoms  0 (ref)  ≥1 |  |  | 1.0  **0.87^a^** | **0.85-0.90** | 1.0  **0.87^a^** | **0.85-0.90** | 1.0  **0.87^a^** | **0.85-0.90** | 1.0  **0.87^a^** | **0.85-0.90** |
| # Chronic diseases  0 (ref)  ≥1 |  |  | 1.0  **0.85^a^** | **0.83-0.87** | 1.0  **0.86^a^** | **0.84-0.89** | 1.0  **0.86^a^** | **0.84-0.89** | 1.0  **0.86^a^** | **0.84-0.89** |
| # GP Contacts  0 (ref)  1-2  ≥3 |  |  | 1.0  0.99  1.03 | 0.95-1.03  0.98-1.07 | 1.0  0.99  1.03 | 0.95-1.03  0.98-1.08 | 1.0  0.99  1.03 | 0.95-1.03  0.98-1.08 | 1.0  0.99  1.03 | 0.95-1.03  0.98-1.08 |
| # of referrals to different specialisms  0 (ref)  1  ≥2 |  |  | 1.0  1.02  1.05 | 0.99-1.06  1.00-1.11 | 1.0  1.02  **1.06^c^** | 0.99-1.05  **1.00-1.11** | 1.0  1.02  **1.06^c^** | 0.99-1.05  **1.00-1.11** | 1.0  1.02  **1.06^c^** | 0.99-1.05  **1.00-1.11** |
| # OOH contacts  0 (ref)  1  ≥2 |  |  | 1.0  0.97  1.05 | 0.94-1.01  0.99-1.12 | 1.0  0.97  1.04 | 0.93-1.01  0.98-1.10 | 1.0  0.97  1.04 | 0.93-1.01  0.98-1.10 | 1.0  0.97  1.04 | 0.93-1.01  0.98-1.10 |
| Demographics |  |  |  |  |  |  |  |  |  |  |
| Gender  Male (ref)  Female |  |  |  |  | 1.0  **0.93^a^** | **0.91-0.95** | 1.0  **0.93^a^** | **0.91-0.95** | 1.0  **0.93^a^** | **0.91-0.95** |
| Age category  ≤11 (ref)  12-15  16-24  25-34  35-44  45-54  55-64  65-74  ≥75 |  |  |  |  | 1.0  0.95  1.02  1.03  1.00  0.99  0.98  **0.92^b^**  **0.94^c^** | 0.90-1.01  0.97-1.07  0.99-1.08  0.95-1.05  0.95-1.04  0.93-1.02  **0.88-0.97**  **0.89-0.99** | 1.0  0.95  1.02  1.03  1.00  1.00  0.98  **0.92^b^**  **0.94^c^** | 0.90-1.01  0.97-1.07  0.99-1.08  0.95-1.05  0.95-1.04  0.93-1.02  **0.87-0.96**  **0.89-0.99** | 1.0  0.95  1.02  1.03  1.00  1.00  0.98  **0.92^b^**  **0.94^c^** | 0.90-1.01  0.98-1.07  0.99-1.08  0.95-1.05  0.95-1.04  0.93-1.02  **0.87-0.96**  **0.89-0.99** |
| Neighborhood characteristics |  |  |  |  |  |  |  |  |  |  |
| Neighborhood SEP  lowest (ref)  below average  average  above average  highest |  |  |  |  |  |  | 1.0  **1.15^a^**  **1.10^a^**  1.04  1.03 | **1.10-1.21**  **1.05-1.15**  1.00-1.09  0.97-1.09 | 1.0  **1.15^a^**  **1.10^a^**  1.04  1.03 | **1.10-1.21**  **1.05-1.15**  1.00-1.09  0.97-1.10 |
| Degree of urbanization  extremely (ref)  strongly  moderately  hardly  not |  |  |  |  |  |  | 1.0  1.06  1.00  **1.08^c^**  0.99 | 1.00-1.12  0.94-1.07  **1.01-1.16**  0.92-1.08 | 1.0  **1.06^c^**  1.00  **1.08^c^**  0.99 | **1.00-1.12**  0.94-1.07  **1.01-1.16**  0.92-1.07 |
| Area deprivation status (postal code)  Not deprived (ref)  Deprived |  |  |  |  |  |  | 1.0  **1.22^a^** | **1.09-1.36** | 1.0  **1.22^a^** | **1.09-1.36** |
| Practice-level characteristics |  |  |  |  |  |  |  |  |  |  |
| EHR system  A (ref)  B |  |  |  |  |  |  |  |  | 1.0  1.57 | 0.62-3.98 |
| Practice type  Single-handed (ref)  Duo  Group |  |  |  |  |  |  |  |  | 1.0  1.14  0.76 | 0.41-3.20  0.24-2.42 |
| Practice size  Small (ref)  Medium  Large |  |  |  |  |  |  |  |  | 1.0  0.76  **3.22^c^** | 0.31-1.83  **1.06-9.81** |
| Random Effects  Intercept var.  ICC | 1.75  0.35 |  | 1.77  0.35 |  | 1.78  0.35 |  | 1.77  0.35 |  | 1.39  0.30 |  |
| Model fit  Log likelihood | -96,609.75 |  | -95,159.27 |  | -95,123.23 |  | -95,077.65 |  | -95,072.13 |  |
| N citizens  N practices | 175,230  46 |  | 175,230  46 |  | 175,230  46 |  | 175,230  46 |  | 175,230  46 |  |

^a^P<0.001; ^b^P<0.01; ^c^P<0.05; ref, reference; var, variance. P-values below 0.05 are considered statistically significant and are marked in bold.

#### Additional file III: Table 2 – Results multilevel logistic regression analysis (2018), n=167,980 citizens from 42 GP practices

|  | Null model | | Model 1 | | Model 2 | | Model 3 | | Model 4 | |
| --- | --- | --- | --- | --- | --- | --- | --- | --- | --- | --- |
|  | **OR** | **95% CI** | **OR** | **95% CI** | **OR** | **95% CI** | **OR** | **95% CI** | **OR** | **95% CI** |
| Overall outcome | **0.38^a^** | **0.26-0.56** |  |  |  |  |  |  |  |  |
| Health and healthcare use |  |  |  |  |  |  |  |  |  |  |
| # of different main group prescriptions  0 (ref)  1  2 or 3  ≥4 |  |  | 1.0  **0.79^a^**  **0.64^a^**  **0.46^a^** | **0.76-0.81**  **0.61-0.66**  **0.44-0.49** | 1.0  **0.79^a^**  **0.66^a^**  **0.50^a^** | **0.77-0.82**  **0.63-0.68**  **0.48-0.53** | 1.0  **0.79^a^**  **0.66^a^**  **0.50^a^** | **0.77-0.82**  **0.63-0.68**  **0.48-0.53** | 1.0  **0.79^a^**  **0.66^a^**  **0.50^a^** | **0.77-0.82**  **0.63-0.68**  **0.48-0.53** |
| # Disease episodes  0 (ref)  1 or 2  ≥3 |  |  | 1.0  0.98  **0.89^b^** | 0.93-1.04  **0.83-0.96** | 1.0  0.97  **0.89^b^** | 0.92-1.03  **0.83-0.96** | 1.0  0.97  **0.89^b^** | 0.92-1.03  **0.83-0.96** | 1.0  0.97  **0.89^b^** | 0.92-1.03  **0.83-0.96** |
| # Acute symptoms  0 (ref)  ≥1 |  |  | 1.0  0.99 | 0.95-1.03 | 1.0  0.99 | 0.95-1.03 | 1.0  0.99 | 0.95-1.03 | 1.0  0.99 | 0.95-1.03 |
| # Long-lasting symptoms  0 (ref)  ≥1 |  |  | 1.0  **0.87^a^** | **0.84-0.89** | 1.0  **0.87^a^** | **0.85-0.90** | 1.0  **0.87^a^** | **0.85-0.90** | 1.0  **0.87^a^** | **0.85-0.90** |
| # Chronic diseases  0 (ref)  ≥1 |  |  | 1.0  **0.81^a^** | **0.79-0.83** | 1.0  **0.84^a^** | **0.81-0.87** | 1.0  **0.84^a^** | **0.81-0.87** | 1.0  **0.84^a^** | **0.81-0.87** |
| # GP Contacts  0 (ref)  1-2  ≥3 |  |  | 1.0  1.02  **1.06^c^** | 0.97-1.06  **1.00-1.11** | 1.0  1.01  **1.06^c^** | 0.97-1.06  **1.01-1.11** | 1.0  1.02  **1.06^c^** | 0.97-1.06  **1.01-1.11** | 1.0  1.02  **1.06^c^** | 0.97-1.06  **1.01-1.11** |
| # of referrals to different specialisms  0 (ref)  1  ≥2 |  |  | 1.0  0.99  0.99 | 0.95-1.02  0.94-1.05 | 1.0  0.99  1.00 | 0.95-1.02  0.94-1.06 | 1.0  0.99  1.00 | 0.95-1.02  0.94-1.06 | 1.0  0.99  1.00 | 0.95-1.02  0.94-1.06 |
| # OOH contacts  0 (ref)  1  ≥2 |  |  | 1.0  1.01  **1.08^c^** | 0.97-1.06  **1.00-1.16** | 1.0  1.00  1.05 | 0.95-1.04  0.98-1.14 | 1.0  1.00  1.05 | 0.95-1.04  0.98-1.13 | 1.0  1.00  1.05 | 0.95-1.04  0.98-1.13 |
| Demographics |  |  |  |  |  |  |  |  |  |  |
| Gender  Male (ref)  Female |  |  |  |  | 1.0  **0.89^a^** | **0.87-0.92** | 1.0  **0.90^a^** | **0.87-0.92** | 1.0  **0.90^a^** | **0.87-0.92** |
| Age category  ≤11 (ref)  12-15  16-24  25-34  35-44  45-54  55-64  65-74  ≥75 |  |  |  |  | 1.0  **0.92^b^**  **1.05^c^**  1.01  0.97  0.99  **0.95^c^**  **0.81^a^**  **0.84^a^** | **0.87-0.98**  **1.00-1.10**  0.96-1.06  0.92-1.01  0.95-1.04  **0.91-1.00**  **0.77-0.85**  **0.80-0.89** | 1.0  **0.92^b^**  **1.05^c^**  1.00  0.97  0.99  **0.95^c^**  **0.81^a^**  **0.84^a^** | **0.87-0.98**  **1.00-1.10**  0.95-1.05  0.92-1.01  0.95-1.04  **0.91-1.00**  **0.77-0.85**  **0.79-0.89** | 1.0  **0.92^b^**  **1.05^c^**  1.00  0.97  0.99  **0.95^c^**  **0.81^a^**  **0.84^a^** | **0.87-0.98**  **1.00-1.10**  0.95-1.05  0.92-1.01  0.95-1.04  **0.91-1.00**  **0.77-0.85**  **0.79-0.89** |
| Neighborhood characteristics |  |  |  |  |  |  |  |  |  |  |
| Neighborhood SEP  lowest (ref)  below average  average  above average  highest |  |  |  |  |  |  | 1.0  **1.09^a^**  0.99  0.99  1.01 | **1.04-1.15**  0.94-1.04  0.95-1.04  0.96-1.07 | 1.0  **1.09^a^**  0.99  0.99  1.01 | **1.04-1.15**  0.94-1.05  0.95-1.04  0.96-1.07 |
| Degree of urbanization  extremely (ref)  strongly  moderately  hardly  not |  |  |  |  |  |  | 1.0  0.97  **0.87^a^**  0.98  0.94 | 0.91-1.03  **0.82-0.93**  0.92-1.06  0.87-1.02 | 1.0  0.97  **0.87^a^**  0.98  0.94 | 0.91-1.03  **0.82-0.93**  0.91-1.05  0.87-1.02 |
| Area deprivation status (postal code)  Not deprived (ref)  Deprived |  |  |  |  |  |  | 1.0  1.09 | 0.99-1.20 | 1.0  1.09 | 0.99-1.20 |
| Practice-level characteristics |  |  |  |  |  |  |  |  |  |  |
| EHR system  A (ref)  B |  |  |  |  |  |  |  |  | 1.0  2.31 | 0.90-5.97 |
| Practice type  Single-handed (ref)  Duo  Group |  |  |  |  |  |  |  |  | 1.0  1.08  1.26 | 0.41-2.84  0.40-3.95 |
| Practice size  Small (ref)  Medium  Large |  |  |  |  |  |  |  |  | 1.0  0.51  1.67 | 0.20-1.28  0.60-4.60 |
| Random Effects  Intercept var.  ICC | 1.58  0.32 |  | 1.62  0.33 |  | 1.63  0.33 |  | 1.62  0.33 |  | 1.22  0.27 |  |
| Model fit  Log likelihood | -90,218.67 |  | -87,926.01 |  | -87,812.34 |  | -87,783.76 |  | -87,777.81 |  |
| N citizens  N practices | 167,380  42 |  | 167,380  42 |  | 167,380  42 |  | 167,380  42 |  | 167,380  42 |  |

^a^P<0.001; ^b^P<0.01; ^c^P<0.05; ref, reference; var, variance. P-values below 0.05 are considered statistically significant and are marked in bold.

## Additional file IV – Flowchart for study sample selection, 2017-2019

#### Additional file IV: Figure 1 – Flowchart for study sample selection: inclusion and exclusion criteria (2017)

**Practices with complete patient consent data**

n = 247 practices; n = 1,016,165 patients

**Excluded: practices having ≥1 patients with missing consent data**

n = 50 practices; n = 229,183 patients

**Patients registered at the same general practice for the entire year**

n = 326 practices; n = 1,223,587 patients

**Excluded: patients not registered at the same general practice for the entire year**

n = 94,956 patients

**Nivel-PCD patient consent data from general practices**

n = 297 practices; n = 1,245,348 patients

**Nivel-PCD practice- and patient-level (demographic and neighborhood characteristics) data from general practices**

N = 326 practices; N = 1,318,543 patients

**Excluded: practices and patients not matching with patient consent dataset**

n = 132 practices; n = 485,575 patients

**Practice-, patient-level (demographic and neighborhood characteristics) and patient consent dataset from general practices**

n = 194 practices; n = 738,012 patients

**Excluded: practice- and patient-level healthcare use and primary care OOH services contact data not matching with practice-, patient-level (demographic and neighborhood) and patient consent dataset**

n = 0 practices; n = 7 patients

**Details of missing practice- or patient-level data for multilevel analysis**

Missing referral data: n = 489,322 patients

Missing OOH services contact data: n = 220,947 patients

Missing practice type data: n = 13,107 patients

Missing degree of urbanization data: n = 12,669 patients

Missing neighborhood SES data: n = 3,833 patients

No variance in consent for 1 EHR system: n = 41,933 patients

**Practice-, patient-level and patient consent dataset from general practices**

n = 194 practices; n = 738,005 patients

**Excluded: practices and patients with missing practice- or patient-level data**

n = 148 practices; n = 562,775 patients

**Final study sample for multilevel analysis**

n = 46 practices; n = 175,230 patients

#### Additional file IV: Figure 2 – Flowchart for study sample selection: inclusion and exclusion criteria (2018)

**Practices with complete patient consent data**

n = 253 practices; n = 1,084,168 patients

**Excluded: practices having ≥1 patients with missing consent data**

n = 50 practices; n = 239,443 patients

**Patients registered at the same general practice for the entire year**

n = 296 practices; n = 1,113,876 patients

**Excluded: patients not registered at the same general practice for the entire year**

n = 0 practices; n = 84,001 patients

**Nivel-PCD patient consent data from general practices**

n = 303 practices; n = 1,323,601 patients

**Nivel-PCD practice- and patient-level (demographic and neighborhood characteristics) data from general practices**

N = 296 practices; N = 1,197,877 patients

**Excluded: practices and patients not matching with patient consent dataset**

n = 101 practices; n = 372,840 patients

**Practice-, patient-level (demographic and neighborhood characteristics) and patient consent dataset from general practices**

n = 195 practices; n = 741,036 patients

**Excluded: practice- and patient-level healthcare use and primary care OOH services contact data not matching with practice-, patient-level (demographic and neighborhood) and patient consent dataset**

n = 0 practices; n = 4 patients

**Practice-, patient-level and patient consent dataset from general practices**

n = 195 practices; n = 741,032 patients

**Details of missing practice- or patient-level data for multilevel analysis**

Missing referral data: n = 551,274 patients

Missing OOH services contact use data: n = 221,311 patients

Missing practice type data: n = 10,095 patients

Missing degree of urbanization data: n = 21,734 patients

Missing neighborhood SES data: n = 14,362 patients

Missing age data: n = 1 patient

**Excluded: practices and patients with missing practice- or patient-level data**

n = 153 practices; n = 573,652 patients

**Final study sample for multilevel analysis**

n = 42 practices; n = 167,380 patients

#### Additional file IV: Figure 3 – Flowchart for study sample selection: inclusion and exclusion criteria (2019)

**Practices with complete patient consent data**

n = 288 practices; n = 1,293,639 patients

**Excluded: practices having ≥1 patients with missing consent data**

n = 155 practices; n = 717,069 patients

**Patients registered at the same general practice for the entire year**

n = 346 practices; n = 1,326,573 patients

**Excluded: patients not registered at the same general practice for the entire year**

n = 0 practices; n = 96,994 patients

**Nivel-PCD patient consent data from general practices**

n = 443 practices; n = 2,010,708 patients

**Nivel-PCD practice- and patient-level (demographic and neighborhood characteristics) data from general practices**

N = 346 practices; N = 1,423,567

**Excluded: practices and patients not matching with patient consent dataset**

n = 101 practices; n = 485,666 patients

**Practice-, patient-level (demographic and neighborhood characteristics) and patient consent dataset from general practices**

n = 225 practices; n = 840,907 patients

**Excluded: practice- and patient-level healthcare use and primary care OOH services contact data not matching with practice-, patient-level (demographic and neighborhood) and patient consent dataset**

n = 0 practices; n = 16 patients

**Practice-, patient-level and patient consent dataset from general practices**

n = 225 practices; n = 840,891 patients

**Details of missing practice- or patient-level data for multilevel analysis**

Missing referral data: n = 540,506 patients

Missing OOH services contact use data: n = 306,691 patients

Missing practice type data: n = 9,729 patients

Missing degree of urbanization data: n = 1,582 patients

Missing neighborhood SES data: n = 5,505 patients

**Excluded: practices and patients with missing practice- or patient-level data**

n = 137 practices; n = 577,832 patients

**Final study sample for multilevel analysis**

n = 65 practices; n = 263,059 patients
